# Supplementary figures and images for: Vitamin D inhibits osteosarcoma by reprogramming nonsense-mediated RNA decay and SNAI2-mediated epithelial-to-mesenchymal transition
Source: Front Oncol. 2023 May 9;13:1188641. doi: 10.3389/fonc.2023.1188641 (PMC10203545; doi:10.3389/fonc.2023.1188641)

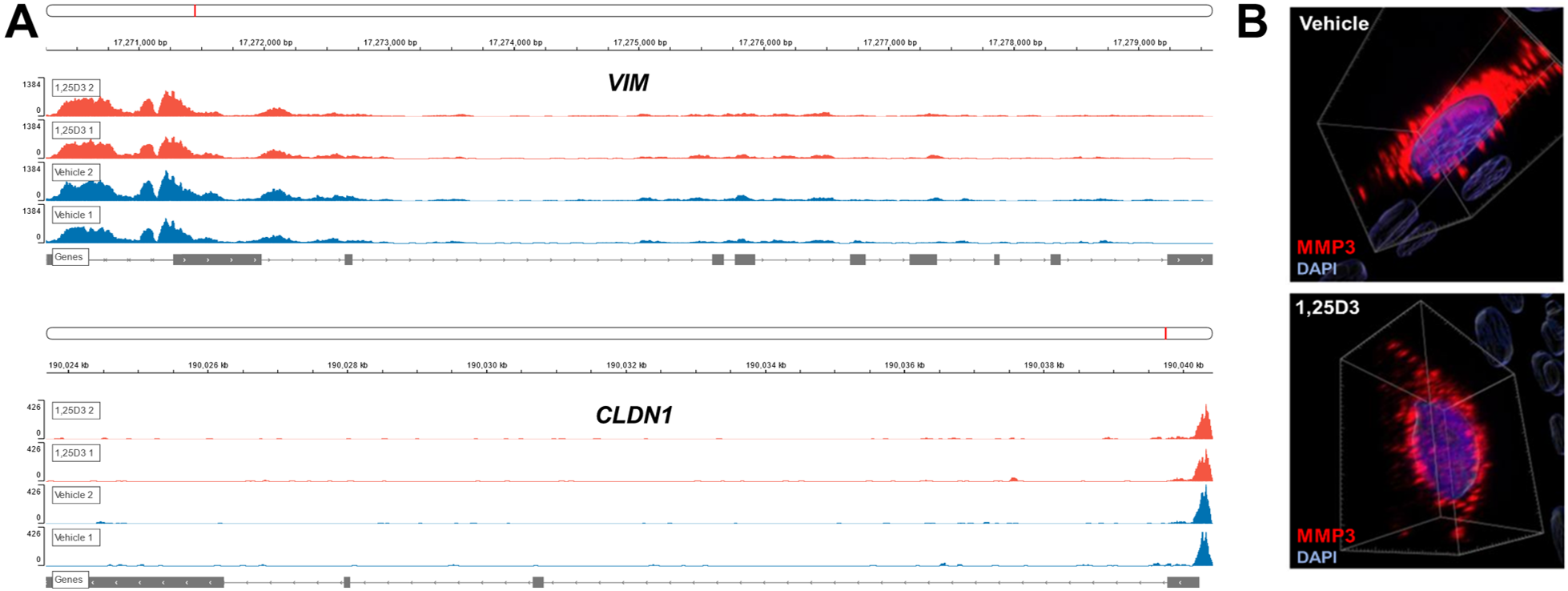

Supplement: Supplementary Figure 1 — MMP13 expression and ATAC-seq genome browser tracks from VIM and CLDN1. (A) 24 hours of 1,25(OH)2D treatment of MG63 cells at 10nM concentration. (B) MG63 and MMP3 expression. Visualized using IMARIS. [file Image_1.tif]

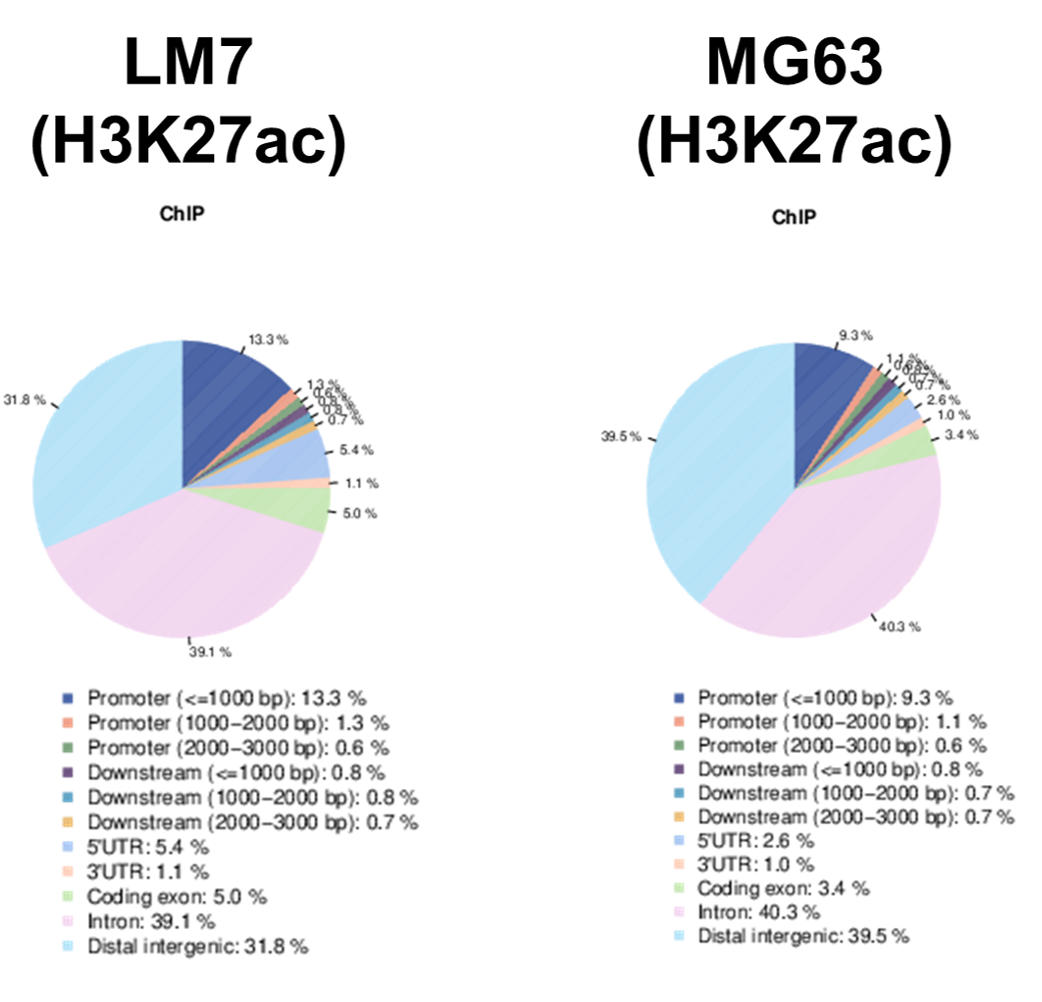

Supplement: Supplementary Figure 2 — Pie chart distribution of ChIP regions (H3K27ac) over chromosomes for LM7 and MG63 osteosarcoma lines. The percentages indicate the total ChIP, with the proportion of ChIP regions indicating the enrichment and significance of genomic structures such as promoters, gene bodies, etc. [file Image_2.tif]

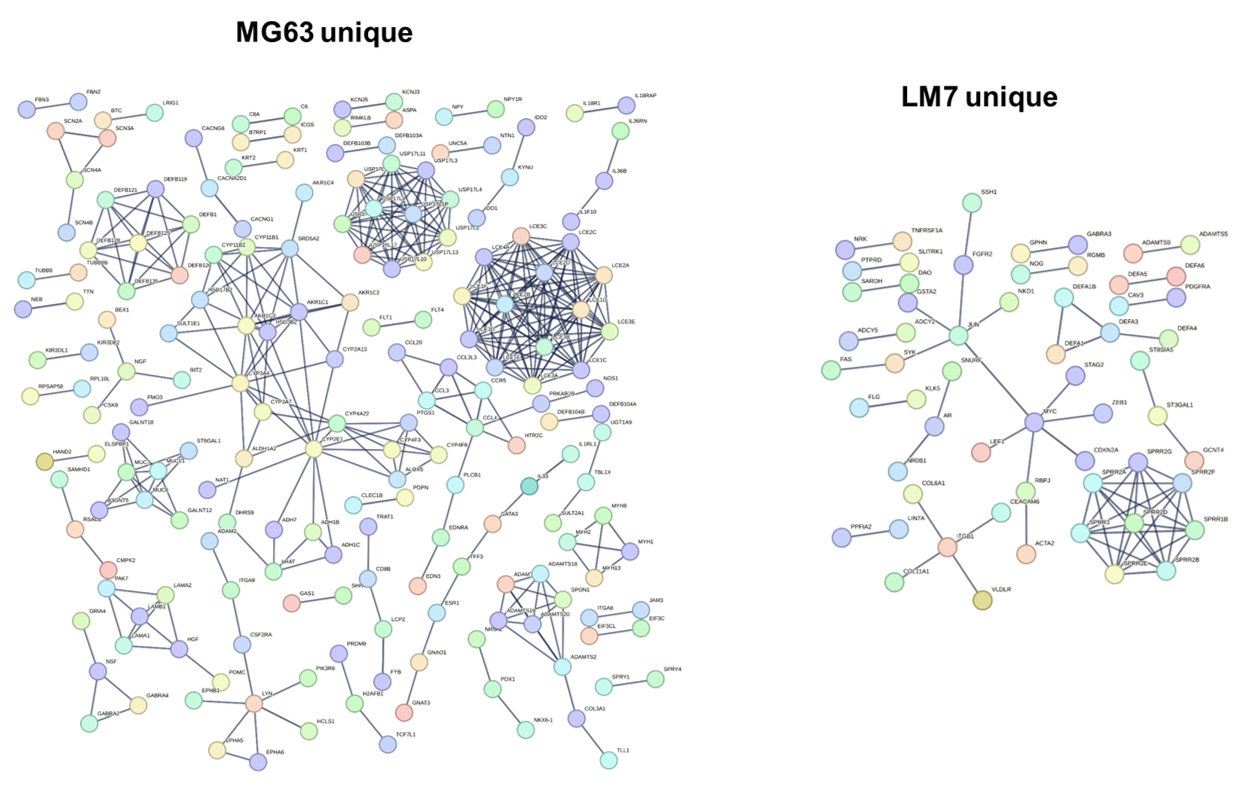

Supplement: Supplementary Figure 3 — Network associate of putative target genes from H3K27ac peaks in MG63 and LM7 osteosarcoma cells. The network corresponds to in the main text. Additional functional enrichment analysis of these networks is provided as Supplementary Material. [file Image_3.tif]

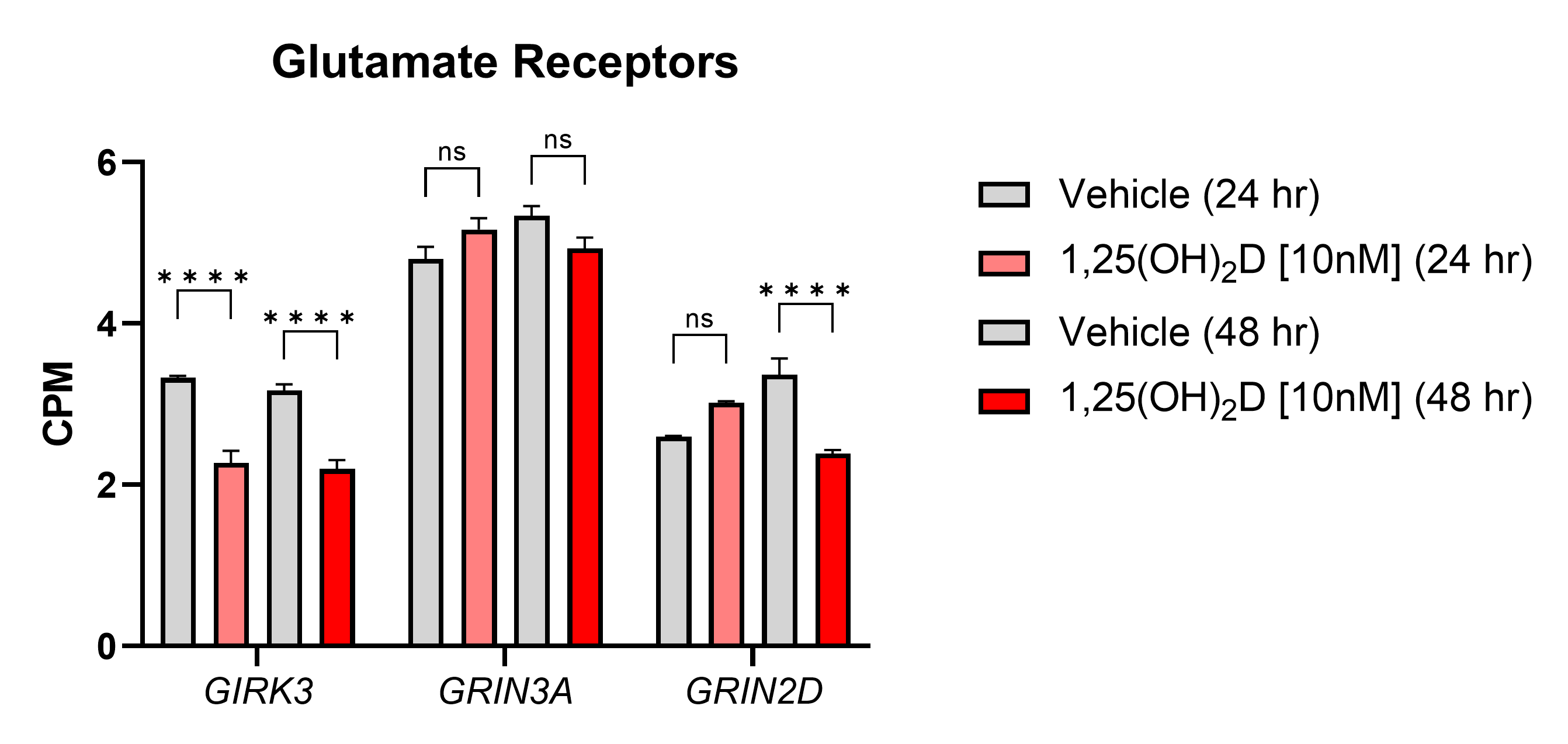

Supplement: Supplementary Figure 4 — Vitamin D regulation of glutamate receptors in osteosarcomas. Glutamate receptor expression in MG63 cells as determined by RNA-seq. Two-way ANOVA Tukey’s multiple comparison test; p ≤ ****0.0001 (n=1). [file Image_4.tif]

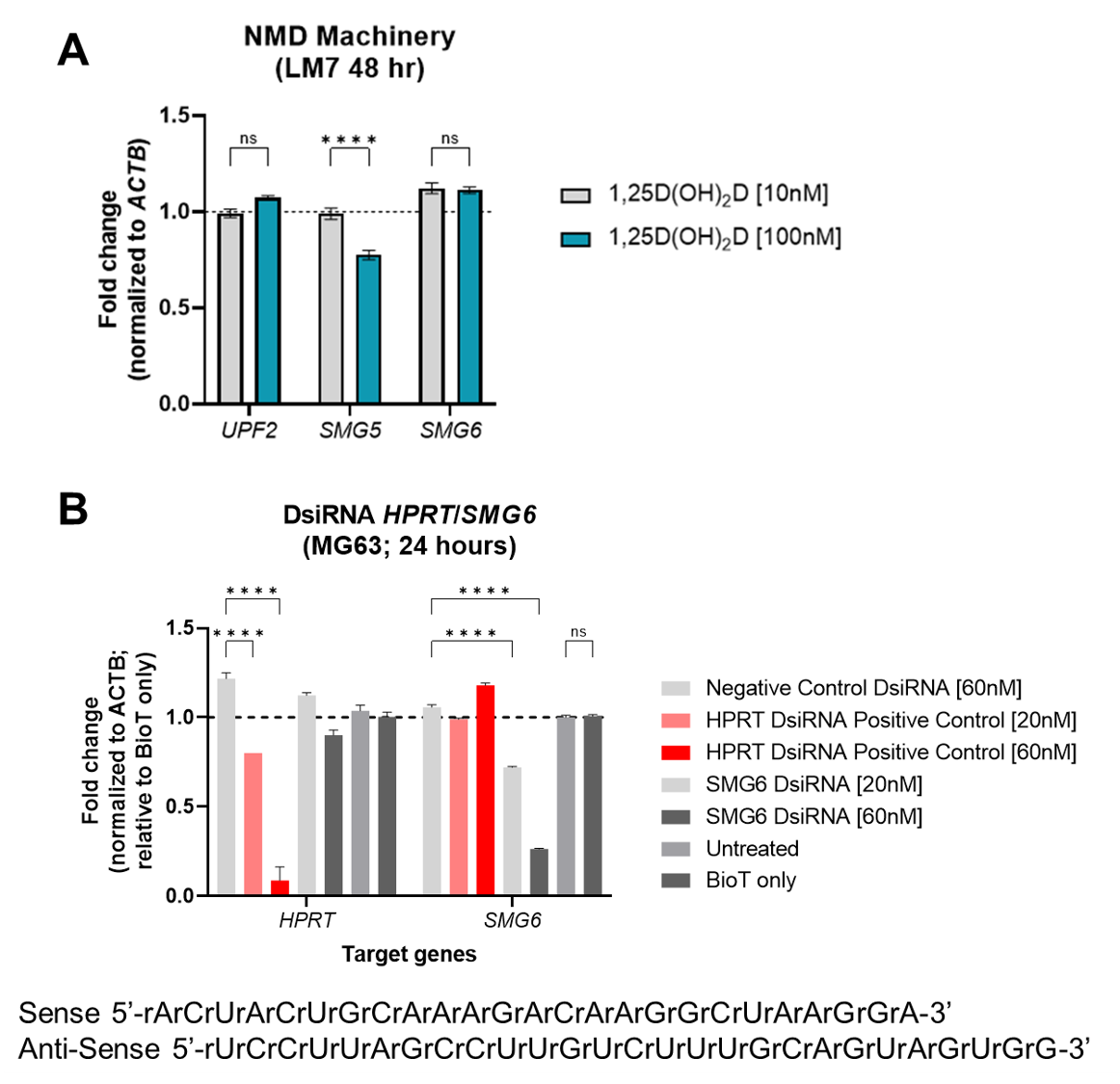

Supplement: Supplementary Figure 5 — Potential NMD target genes modulated by 1,25(OH)2D in MG63 cells. Four major studies that manipulated the NMD machinery were screened for common NMD target and non-target genes. Those genes were then compared to differentially regulated gene sets modulated by 1,25(OH)2D. The overlapping gene set expression pattern was analyzed in the main text. [file Image_5.tif]

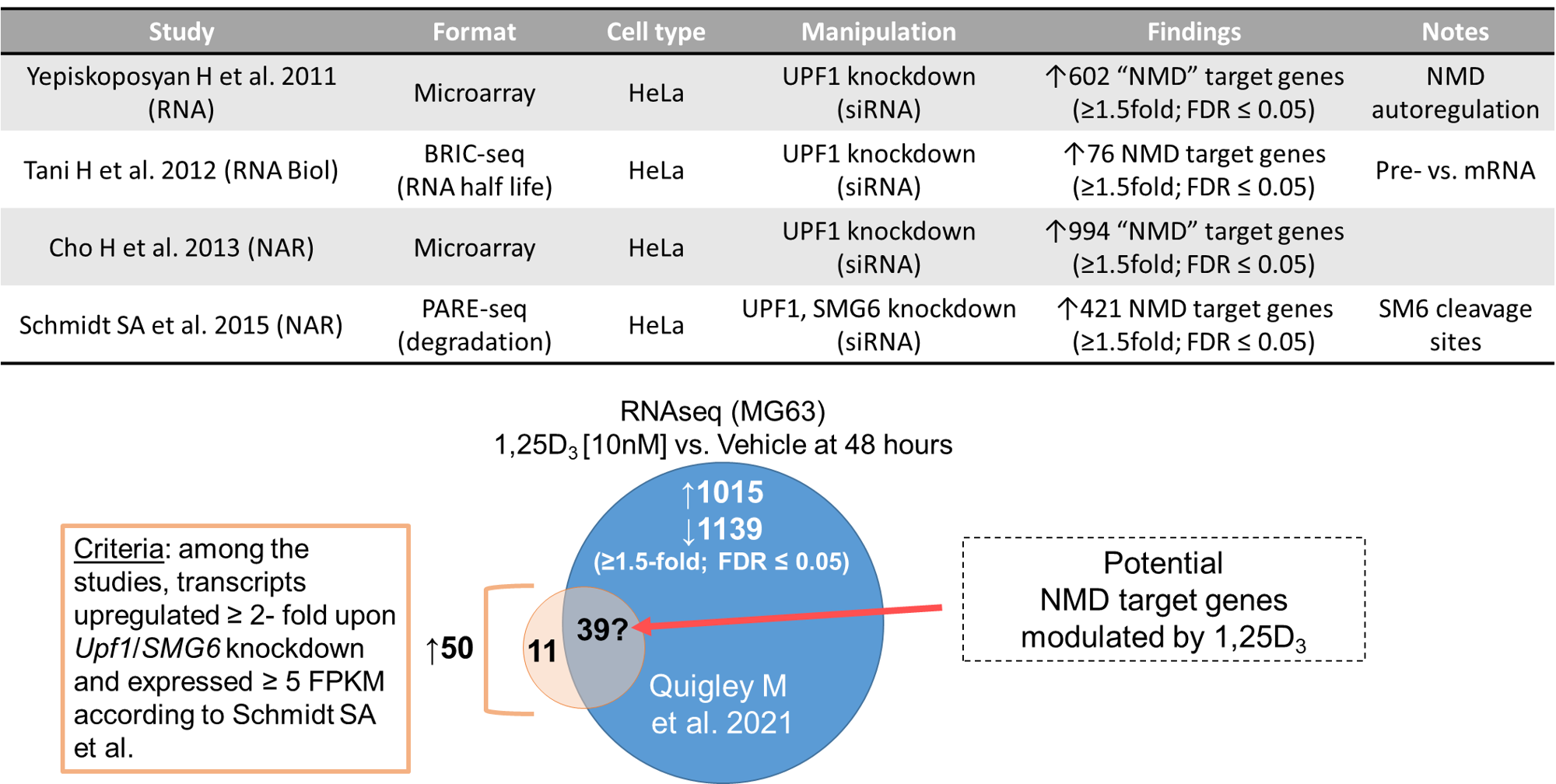

Supplement: Supplementary Figure 6 — DsiRNA knockdown of control HPRT and SMG6 in MG63 cells. (A) Partial suppression by 1,25(OH)2D of the NMD machinery genes in LM7 cells. LM7 cells treated for 48 hours with 100nM 1,25(OH)2D exhibit a downregulation of SMG5. Two-way ANOVA Tukey’s test for multiple comparisons; p ≤ ****0.0001 (n=3). (B) Specific knockdown of SMG6 using the SMG6.13.3 duplex. Duplex sequences shown below the graph. Two-way ANOVA test with Tukey’s multiple comparisons. All data represent the mean standard deviation (S.D.) of at least three separate experiments. ****p < 0.0001. [file Image_6.tif]

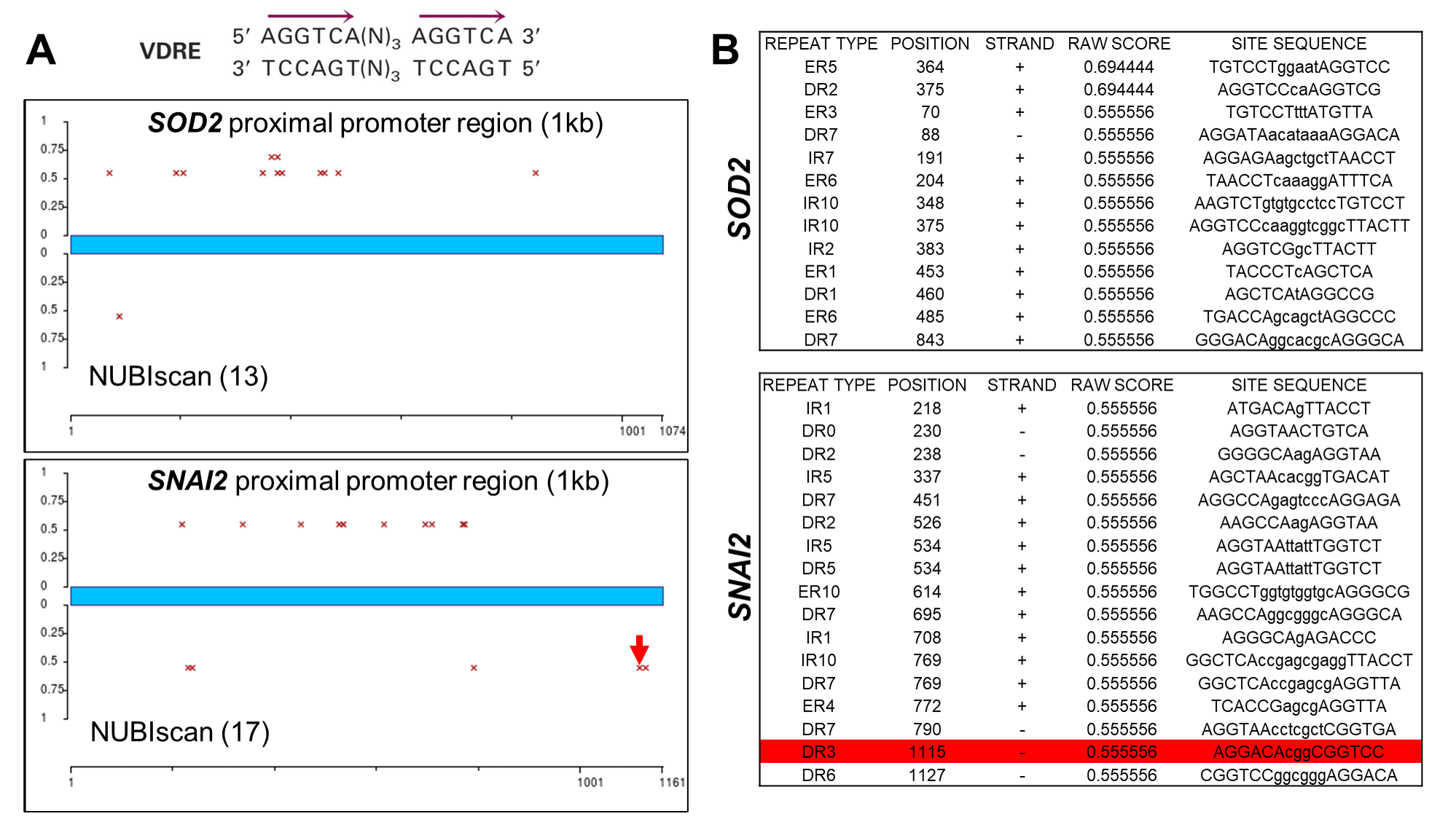

Supplement: Supplementary Figure 7 — VDR directly enhances the transcriptional activity of SNAI2 but not SOD2. (A) Using NUBIScan (http://nubiscan.unibas.ch), potential vitamin D receptor response elements (VDREs) in the SOD2 and SNAI2 promoter regions were anticipated. As shown by the blue bar, the starting point is the transcription start sites (TSS, +1000bp). (B) SOD2 has 13 nuclear receptor binding sites, while SNAI2 had 17. SNAI2 was the only gene that featured a putative VDRE with a direct repeat 3 (DR3) intervals for RXR binding (highlighted by the red arrow and bar). [file Image_7.tif]

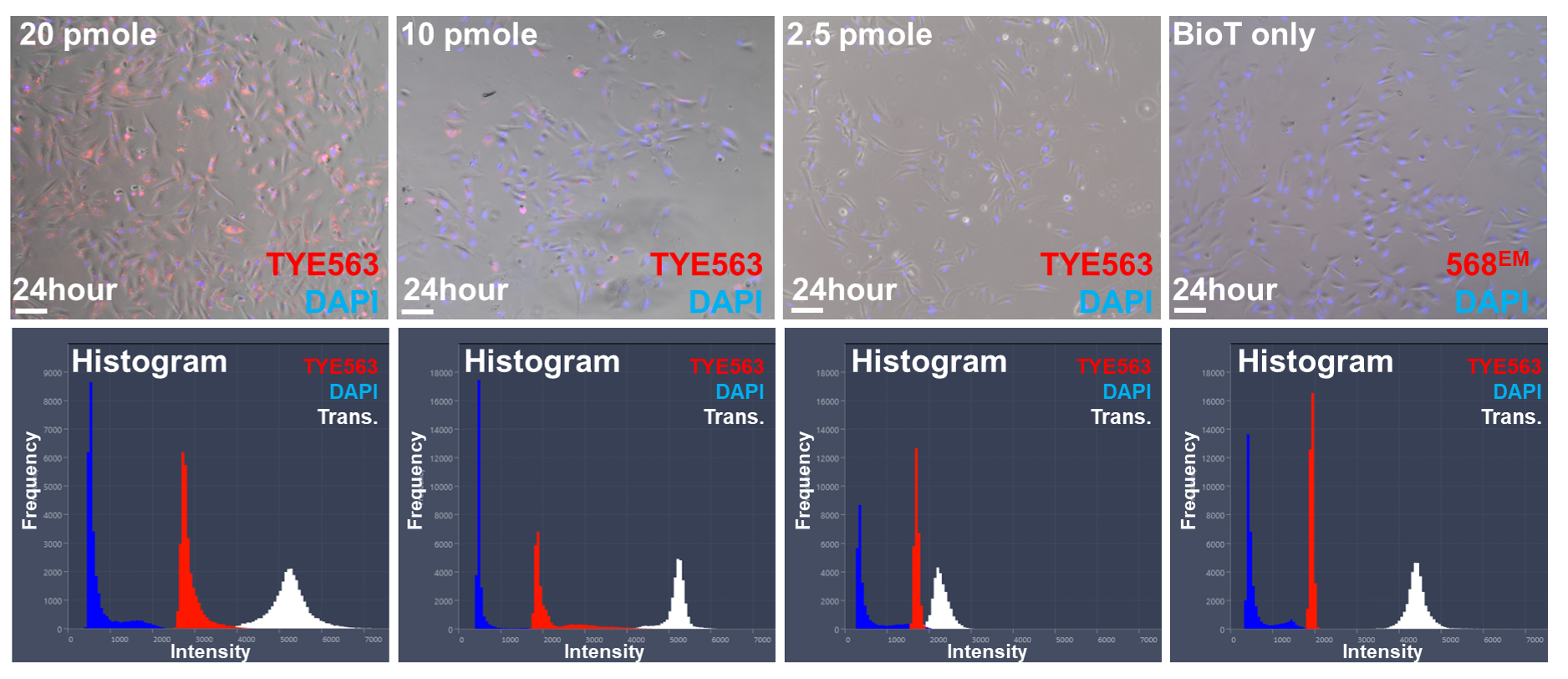

Supplement: Supplementary Figure 8 — Transfection efficiency of DsiRNA Trifecta reagents in LM7 cells. After 24 hours of incubation, the transfection efficiency of 20pmole of TYE563-labeled DsiRNA was greater than 90%. Bar = 100µm Below: Histograms revealed that the 20pmole concentration comprised a bigger fraction of cells with a higher intensity. [file Image_8.tif]

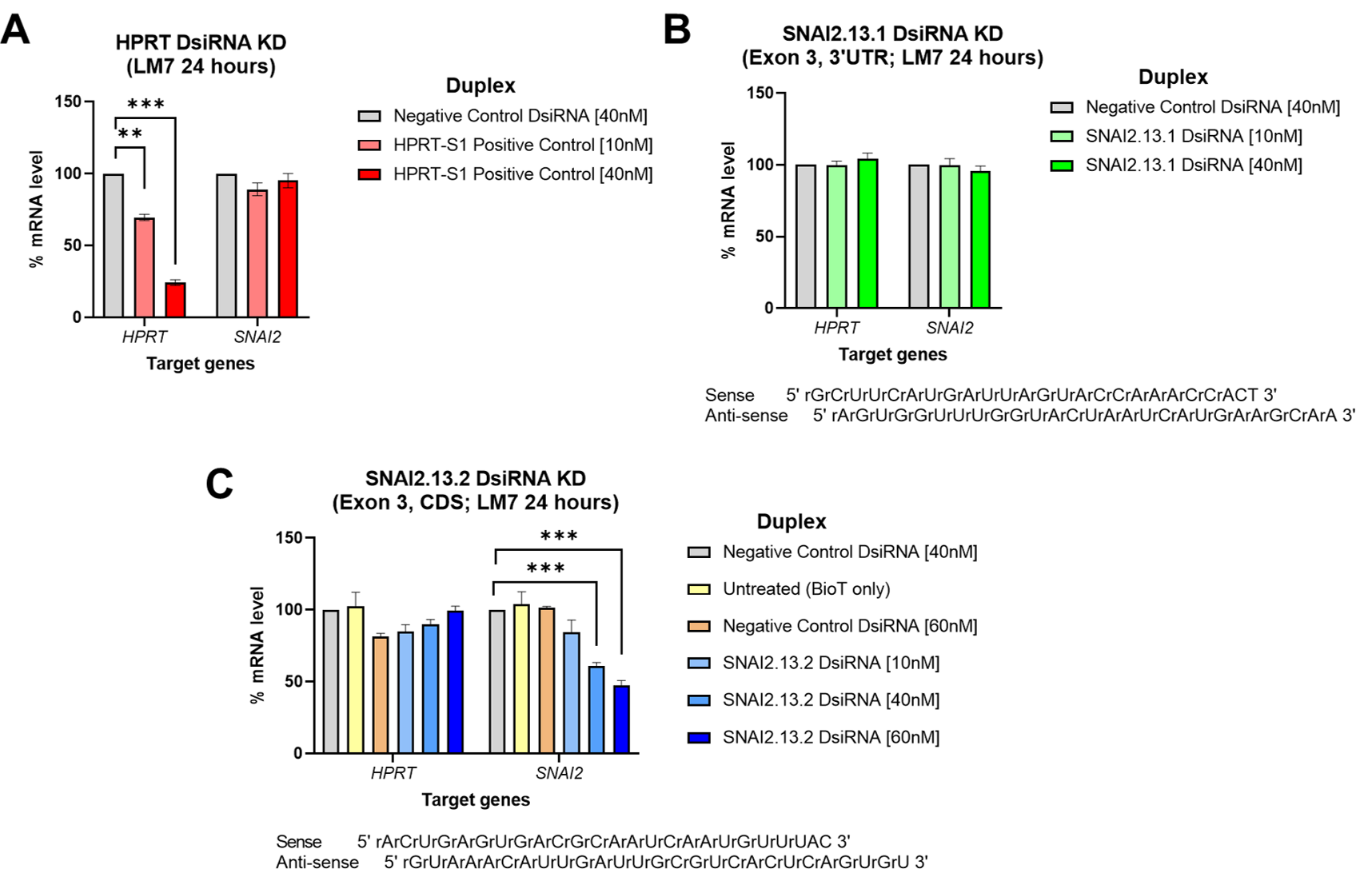

Supplement: Supplementary Figure 9 — DsiRNA knockdown of SNAI2 and control HPRT in LM7 cells (A) HPRT cleavage by DsiRNA. (B) Using the SNAI2.13.1 duplex, DsiRNA aimed to inhibit SNAI2 expression. (C) Successful SNAI2 DsiRNA knockdown with the SNAI2.13.2 duplex. [file Image_9.tif]

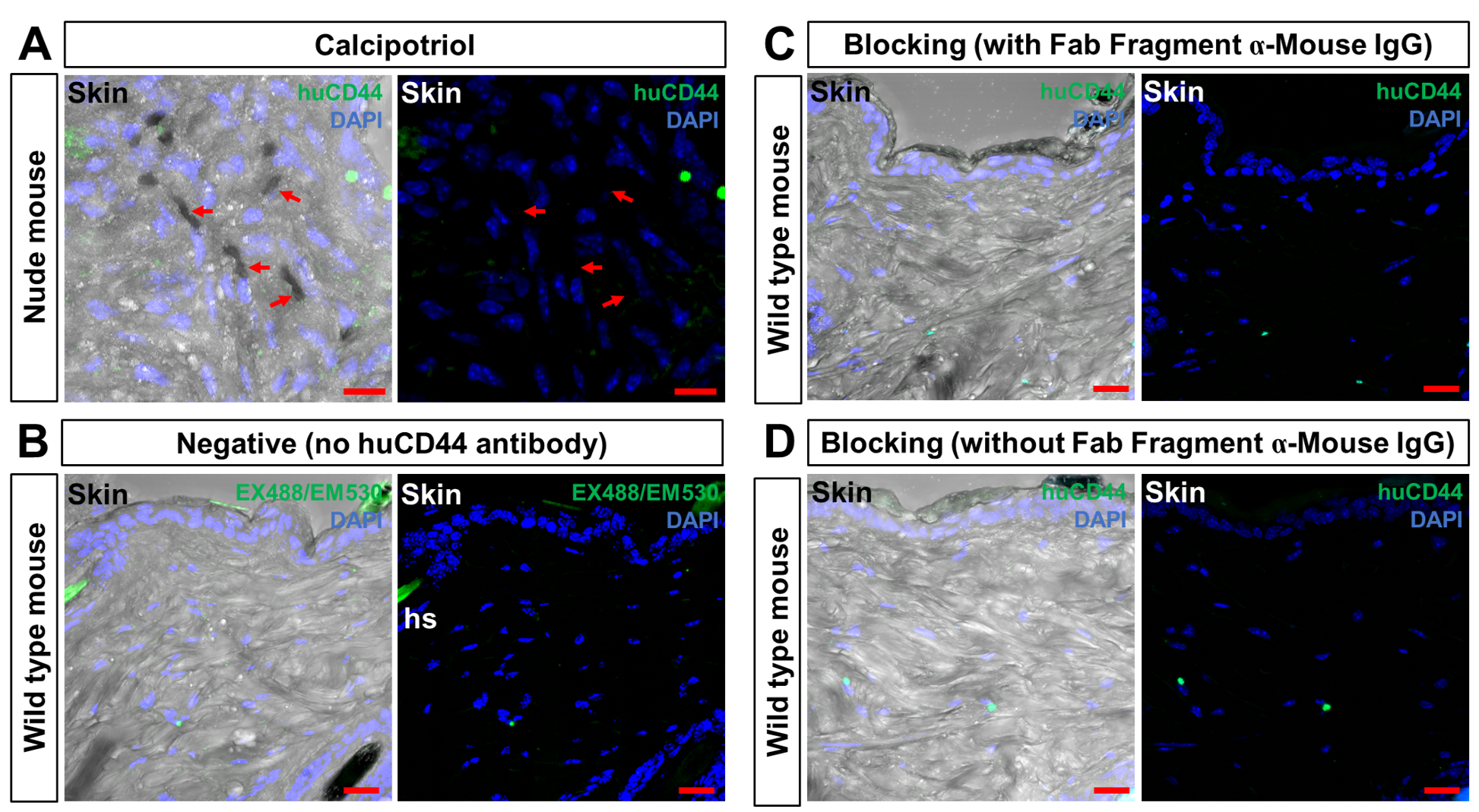

Supplement: Supplementary Figure 10 — huCD44 antibody testing. (A) Application of huCD44 antibody against calcipotriol-treated LM7 xenograft mouse skin. Red arrows depict red blood cells that are negative for huCD44. Bar = 10µm (B) Negative control. Wild type mice skin sections were treated similarly but without huCD44 antibody. Typical autofluorescence of hair shafts (hs) are seen. Bar = 20µm (C) huCD44 background analysis with mouse Fab fragment blocking. Bar = 20µm (D) huCD44 background analysis without mouse Fab fragment blocking. Bar = 20µm [file Image_10.tif]

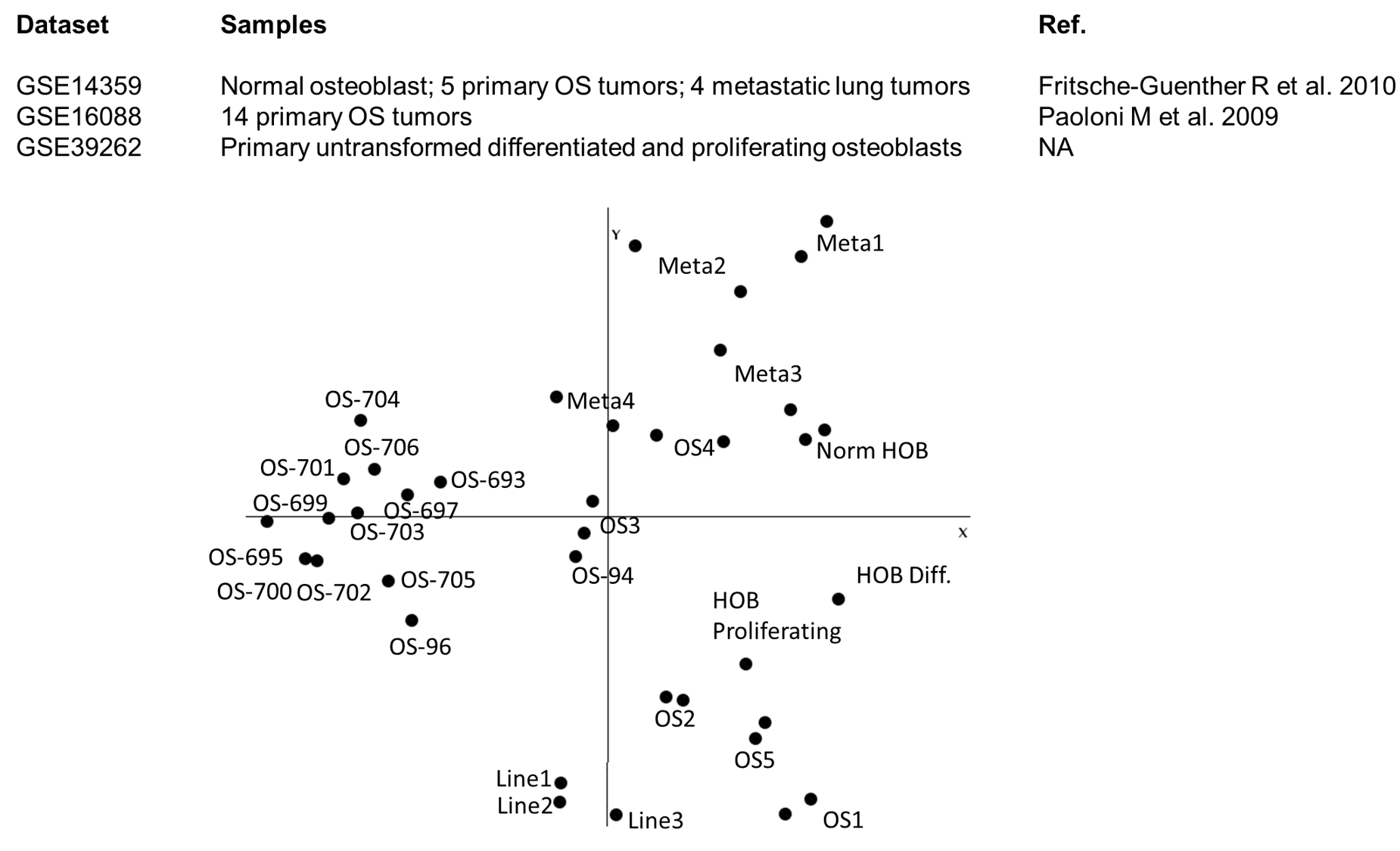

Supplement: Supplementary Figure 11 — Principal component analysis of osteosarcoma and normal samples used for comparative gene expression analysis. The GEO series accession numbers are referenced for each sample set. [file Image_11.tif]

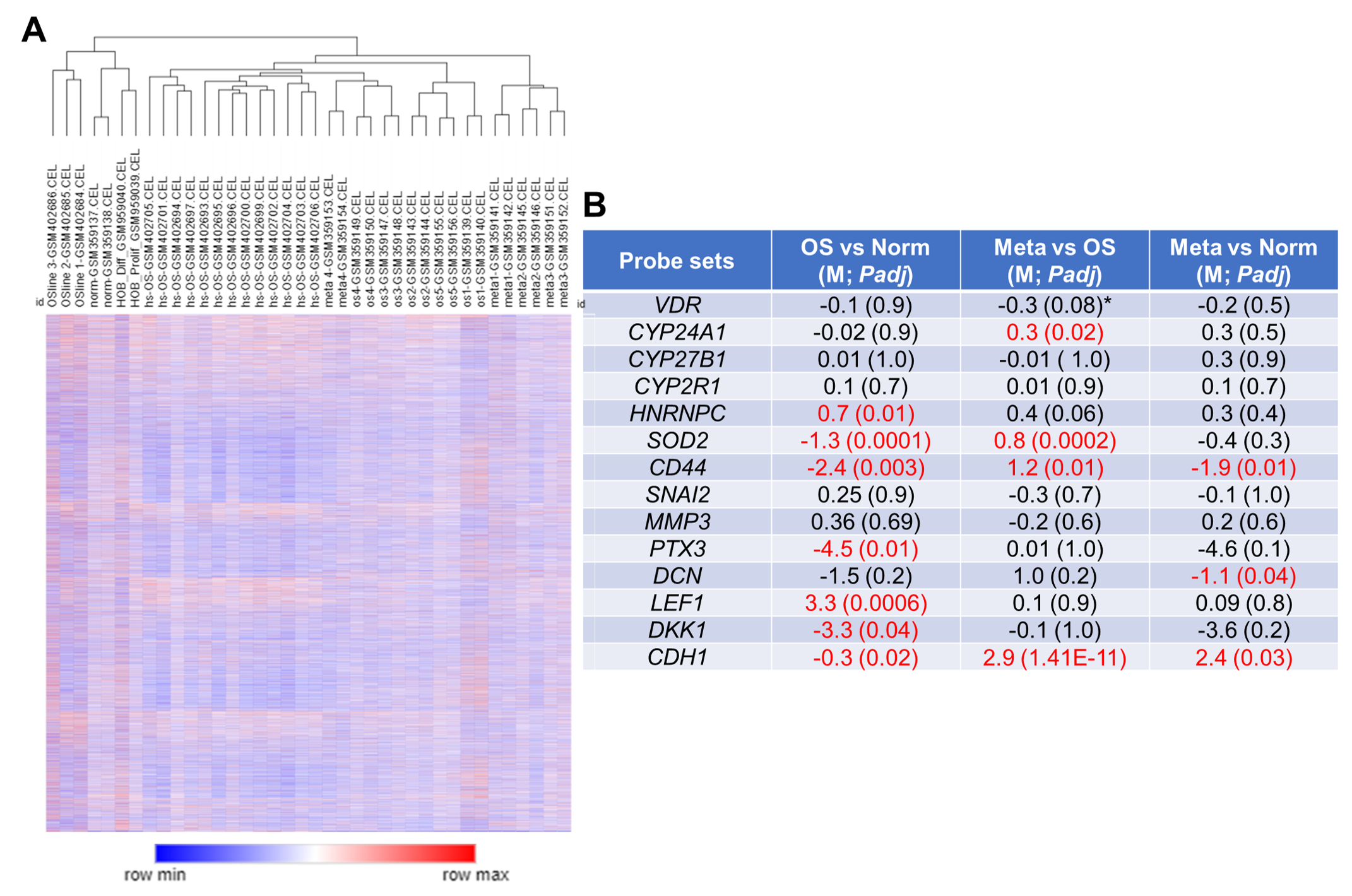

Supplement: Supplementary Figure 12 — Comparative analysis between normal bone tissue, and primary osteosarcoma and metastasized lung tumors. (A) Hierarchical clustering of cDNA arrays of human tumor samples and OS cell lines using Euclidean distance and full linkage. The GEO sample accession number is assigned to each sample (Corresponds to Figure). BioConductor software for traditional 3’ arrays were used for preprocessing and analysis of Affymetrix GeneChip data. RMA (Robust Multiarray Analysis) was utilized for array preparation. Moderated t-statistics (limma) were utilized to identify genes with differential regulation. (B) The chart displays the M value, which is the log-ratio of the probeset intensities, as well as the corrected p values of numerous comparisons. Normal has normal osteoblast cells and normal tissue. Red indicates signatures that statistically differed from the others. [file Image_12.tif]
