# Supplementary material for: Vitamin D inhibits osteosarcoma by reprogramming nonsense-mediated RNA decay and SNAI2-mediated epithelial-to-mesenchymal transition
Source: Front Oncol. 2023 May 9;13:1188641. doi: 10.3389/fonc.2023.1188641 (PMC10203545; doi:10.3389/fonc.2023.1188641)
Supplement: Supplementary File S1 — Functional enrichment annotation analysis of MG63 unique H3K27ac target genes [file DataSheet_1.pdf]

Network Stats

number of nodes:	555
number of edges:	64
average node degree:	0.231
avg. local clustering coefficient:	0.0907
expected number of edges:	24
PPI enrichment p-value:	1.15e-11

Functional enrichments in network

Biological Process (Gene Ontology)

GO-term description	*count in network*	*strength*	*false discovery rate*
GO:0051673 Membrane disruption in other organism	7 of 12	1.31	0.00071
GO:0021889 Olfactory bulb interneuron differentiation	5 of 12	1.17	0.0197
GO:0021772 Olfactory bulb development	8 of 32	0.95	0.0050
GO:0002227 Innate immune response in mucosa	6 of 26	0.91	0.0431
GO:1905606 Regulation of presynapse assembly	7 of 34	0.86	0.0267
GO:0051965 Positive regulation of synapse assembly	13 of 66	0.84	0.00067
GO:0021879 Forebrain neuron differentiation	8 of 46	0.79	0.0256
GO:0050832 Defense response to fungus	8 of 48	0.77	0.0307
GO:0021872 Forebrain generation of neurons	9 of 58	0.74	0.0229
GO:0019731 Antibacterial humoral response			

GO:0009620 Response to fungus	9 of 59	0.73	0.0248
GO:0051963 Regulation of synapse assembly	9 of 61	0.72	0.0284
GO:0070268 Cornification	14 of 106	0.67	0.0032
GO:0007416 Synapse assembly	14 of 113	0.64	0.0050
GO:0035821 Modulation of process of other organism	11 of 96	0.61	0.0381
GO:0021953 Central nervous system neuron differentiation	12 of 115	0.57	0.0431
GO:0007156 Homophilic cell adhesion via plasma membrane adhesion molecules	18 of 183	0.54	0.0051
GO:0098742 Cell-cell adhesion via plasma-membrane adhesion molecules	16 of 164	0.54	0.0120
GO:0031424 Keratinization	24 of 257	0.52	0.0014
GO:0050807 Regulation of synapse organization	20 of 226	0.49	0.0064
GO:0007608 Sensory perception of smell	20 of 228	0.49	0.0068
GO:0030216 Keratinocyte differentiation	33 of 411	0.45	0.00067
GO:0007187 G protein-coupled receptor signaling pathway, coupled to cyclic nucleotide second messenger	21 of 268	0.44	0.0159

GO:0099537 Trans-synaptic signaling	19 of 254	0.42	0.0446
GO:0007268 Chemical synaptic transmission	32 of 436	0.41	0.0024
GO:0098609 Cell-cell adhesion	30 of 418	0.4	0.0041
GO:0050906 Detection of stimulus involved in sensory perception	35 of 505	0.39	0.0024
GO:0007606 Sensory perception of chemical stimulus	35 of 497	0.39	0.0024
GO:0030900 Forebrain development	34 of 484	0.39	0.0024
GO:0061564 Axon development	27 of 387	0.39	0.0115
GO:0043588 Skin development	28 of 421	0.37	0.0159
GO:0030855 Epithelial cell differentiation	25 of 382	0.36	0.0379
GO:0050877 Nervous system process	42 of 673	0.34	0.0025
GO:0051606 Detection of stimulus	80 of 1352	0.32	4.49e-06
GO:0007600 Sensory perception	39 of 659	0.32	0.0088
GO:0007155 Cell adhesion	53 of 923	0.31	0.0024
	53 of 925	0.31	0.0024

**GO:0030182**<br>**Neuron differentiation**	**56 of 1019**	**0.29**	**0.0025**
**GO:0003008**<br>**System process**	**104 of 1942**	**0.28**	**3.03e-06**
**GO:0007186**<br>**G protein-coupled receptor signaling pathway**	**68 of 1255**	**0.28**	**0.00072**
**GO:0009888**<br>**Tissue development**	**88 of 1760**	**0.25**	**0.00067**
**GO:0060429**<br>**Epithelium development**	**56 of 1109**	**0.25**	**0.0115**
**GO:0007417**<br>**Central nervous system development**	**50 of 988**	**0.25**	**0.0238**
**GO:0009887**<br>**Animal organ morphogenesis**	**49 of 967**	**0.25**	**0.0256**
**GO:0048699**<br>**Generation of neurons**	**72 of 1551**	**0.21**	**0.0120**
**GO:0022008**<br>**Neurogenesis**	**74 of 1657**	**0.2**	**0.0248**
**GO:0009653**<br>**Anatomical structure morphogenesis**	**93 of 2165**	**0.18**	**0.0137**
GO:0048869<br>Cellular developmental process	151 of 3757	0.15	0.0025
**GO:0030154**<br>**Cell differentiation**	**149 of 3702**	**0.15**	**0.0026**
**GO:0032501**<br>**Multicellular organismal process**	**270 of 6933**	**0.14**	**2.79e-06**

	count in network	strength	false discovery rate
**GO:0048513** **Animal organ development**	**126 of 3197**	**0.14**	**0.0212**
**GO:0048731** **System development**	**171 of 4426**	**0.13**	**0.0038**
**GO:0048856** **Anatomical structure development**	**200 of 5402**	**0.12**	**0.0051**
**GO:0007275** **Multicellular organism development**	**188 of 5023**	**0.12**	**0.0055**
**GO:0032502** **Developmental process**	**211 of 5841**	**0.11**	**0.0100**

KEGG Pathways

Pathway description	*count in network*	*strength*	*false discovery rate*
hsa00982 Drug metabolism - cytochrome P450	9 of 64	0.7	0.0294
hsa05204 Chemical carcinogenesis	9 of 75	0.63	0.0426
hsa04740 Olfactory transduction	32 of 420	0.43	0.00041
hsa04080 Neuroactive ligand-receptor interaction	22 of 330	0.37	0.0376

Reactome Pathways

Pathway description	*count in network*	*strength*	*false discovery rate*
HSA-1462054 Alpha-defensins			

		5 of 9	1.29	0.0069
HSA-388844	Receptor-type tyrosine-protein phosphatases	6 of 19	1.05	0.0117
HSA-6794362	Protein-protein interactions at synapses	12 of 85	0.7	0.0045
HSA-6809371	Formation of the cornified envelope	15 of 127	0.62	0.0040
HSA-6805567	Keratinization	21 of 210	0.55	0.0014
HSA-381753	Olfactory Signaling Pathway	32 of 391	0.46	0.00062
HSA-418555	G alpha (s) signalling events	38 of 532	0.4	0.00062
HSA-388396	GPCR downstream signalling	58 of 1094	0.27	0.0038
HSA-372790	Signaling by GPCR	60 of 1166	0.26	**0.0041**
