# Supplementary material for: Vitamin D inhibits osteosarcoma by reprogramming nonsense-mediated RNA decay and SNAI2-mediated epithelial-to-mesenchymal transition
Source: Front Oncol. 2023 May 9;13:1188641. doi: 10.3389/fonc.2023.1188641 (PMC10203545; doi:10.3389/fonc.2023.1188641)
Supplement: Supplementary File S2 — Functional enrichment annotation analysis of LM7 unique H3K27ac target genes [file DataSheet_2.pdf]

Network Stats
number of nodes: 1102
number of edges: 367
average node degree: 0.666
avg. local clustering coefficient: 0.142
expected number of edges: 92
PPI enrichment p-value: < 1.0e-16

Functional enrichments in network

Biological Process (Gene Ontology)

GO-term description	*count in network*	*strength*	*false discovery rate*
GO:2001030 Negative regulation of cellular glucuronidation	6 of 8	1.12	0.0097
GO:1904224 Negative regulation of glucuronosyltransferase activity	6 of 8	1.12	0.0097
GO:0052696 Flavonoid glucuronidation	5 of 7	1.1	0.0378
GO:1902644 Tertiary alcohol metabolic process	6 of 13	0.91	0.0471
GO:0009812 Flavonoid metabolic process	6 of 13	0.91	0.0471
GO:0033141 Positive regulation of peptidyl-serine phosphorylation of stat protein	9 of 21	0.88	0.0047
GO:0002323 Natural killer cell activation involved in immune response	10 of 24	0.87	0.0023
GO:0042573 Retinoic acid metabolic process	11 of 32	0.79	0.0033

GO:0002548
Monocyte chemotaxis

14 of 43 0.76 0.00051
GO:0001580
Detection of chemical stimulus involved in sensory perception of bitter taste

12 of 39 0.74 0.0033
GO:0086010
Membrane depolarization during action potential

9 of 30 0.73 0.0279
GO:0050913
Sensory perception of bitter taste

13 of 44 0.72 0.0023
GO:0042100
B cell proliferation

11 of 43 0.66 0.0200
GO:0031424
Keratinization

55 of 226 0.64 1.99e-13
GO:0043330
Response to exogenous dsrna

11 of 46 0.63 0.0294
GO:0048247
Lymphocyte chemotaxis

11 of 50 0.59 0.0471
GO:0030101
Natural killer cell activation

12 of 56 0.58 0.0351
GO:0030216
Keratinocyte differentiation

56 of 268 0.57 1.57e-11
GO:0034308
Primary alcohol metabolic process

17 of 82 0.57 0.0047
GO:0070252
Actin-mediated cell contraction

18 of 90 0.55 0.0042
GO:0031640
Killing of cells of other organism

18 of 91 0.55 0.0047
GO:0050829
Defense response to gram-negative bacterium

19 of 98 0.54 0.0037
GO:0034754
Cellular hormone metabolic process

24 of 125 0.53 0.00051
GO:0009913
Epidermal cell differentiation

59 of 315 0.52 1.28e-10
GO:0061844
Antimicrobial humoral immune response mediated by antimicrobial peptide

21 of 113 0.52 0.0026
GO:0042180
Cellular ketone metabolic process

14 of 76 0.51 0.0420
GO:0120254
Olefinic compound metabolic process

19 of 106 0.5 0.0077
GO:1990869
Cellular response to chemokine

16 of 90 0.5 0.0265
GO:0001508
Action potential

17 of 97 0.49 0.0202
GO:0043588
Skin development

65 of 382 0.48 2.51e-10
GO:0019730
Antimicrobial humoral response

27 of 160 0.48 0.00084

GO:0008544
Epidermis development

69 of 419 0.47 2.02e-10
GO:0006959
Humoral immune response

46 of 275 0.47 1.11e-06
GO:0001906
Cell killing

21 of 126 0.47 0.0077
GO:0006805
Xenobiotic metabolic process

18 of 112 0.46 0.0301
GO:0042445
Hormone metabolic process

31 of 194 0.45 0.00051
GO:0030048
Actin filament-based movement

19 of 121 0.45 0.0278
GO:0097529
Myeloid leukocyte migration

19 of 123 0.44 0.0315
GO:0035637
Multicellular organismal signaling

20 of 137 0.41 0.0403
GO:0060326
Cell chemotaxis

29 of 204 0.4 0.0049
GO:0030855
Epithelial cell differentiation

92 of 673 0.39 2.51e-10
GO:0050907
Detection of chemical stimulus involved in sensory perception

60 of 434 0.39 2.36e-06
GO:0009593
Detection of chemical stimulus

63 of 470 0.38 2.55e-06

GO:0070374

Positive regulation of erk1 and erk2 cascade

28 of 209 0.38 0.0132

GO:0006813

Potassium ion transport

23 of 174 0.37 0.0475

GO:0007606

Sensory perception of chemical stimulus

63 of 484 0.36 4.80e-06

GO:0050906

Detection of stimulus involved in sensory perception

64 of 497 0.36 5.01e-06

GO:0042742

Defense response to bacterium

36 of 277 0.36 0.0035

GO:0007608

Sensory perception of smell

50 of 411 0.33 0.00057

GO:0007187

G protein-coupled receptor signaling pathway, coupled to cyclic nucleotide second messenger

30 of 254 0.32 0.0424

GO:0007186

G protein-coupled receptor signaling pathway

142 of 1255 0.3 9.60e-11

GO:0051606

Detection of stimulus

74 of 659 0.3 4.18e-05

GO:0070372

Regulation of erk1 and erk2 cascade

33 of 292 0.3 0.0433

GO:0050877

Nervous system process

145 of 1352 0.28 1.12e-09

GO:0007600<br>Sensory perception	100 of 923	0.28	2.55e-06
GO:0006935<br>Chemotaxis	57 of 545	0.27	0.0049
GO:0010817<br>Regulation of hormone levels	55 of 524	0.27	0.0057
GO:0003008<br>System process	199 of 1942	0.26	5.75e-12
GO:0060429<br>Epithelium development	114 of 1109	0.26	2.55e-06
GO:0042391<br>Regulation of membrane potential	45 of 440	0.26	0.0351
GO:0098542<br>Defense response to other organism	88 of 900	0.24	0.00055
GO:0006952<br>Defense response	123 of 1296	0.23	2.45e-05
GO:0009888<br>Tissue development	155 of 1760	0.19	3.09e-05
GO:0051707<br>Response to other organism	108 of 1256	0.18	0.0051
GO:0007267<br>Cell-cell signaling	98 of 1145	0.18	0.0117
GO:0034220<br>Ion transmembrane transport			

87 of 1010 0.18 0.0230

GO:0006811
Ion transport

109 of 1344 0.16 0.0279

GO:0032501
Multicellular organismal process

553 of 6933 0.15 2.01e-18

GO:0048513
Animal organ development

254 of 3197 0.15 8.70e-06

GO:0030154
Cell differentiation

290 of 3702 0.14 2.55e-06

GO:0048869
Cellular developmental process

291 of 3757 0.14 5.67e-06

GO:0006928
Movement of cell or subcellular component

118 of 1501 0.14 0.0424

GO:0009605
Response to external stimulus

174 of 2310 0.13 0.0150

GO:0007275
Multicellular organism development

373 of 5023 0.12 2.55e-06

GO:0048731
System development

328 of 4426 0.12 2.78e-05

GO:0048856
Anatomical structure development

396 of 5402 0.11 2.55e-06

GO:0023052
Signaling

381 of 5239 0.11 1.06e-05

GO:0007154<br>Cell communication	383 of 5320	0.11	2.60e-05
GO:0032502<br>Developmental process	417 of 5841	0.1	1.15e-05
GO:0007165<br>Signal transduction	343 of 4876	0.1	0.0013
GO:0042221<br>Response to chemical	308 of 4333	0.1	0.0022
GO:0050896<br>Response to stimulus	522 of 8046	0.06	0.0059

KEGG Pathways

Pathway description	*count in network*	*strength*	*false discovery rate*
**hsa00140**<br>**Steroid hormone biosynthesis**	**20 of 59**	**0.78**	**1.28e-06**
**hsa00053**<br>**Ascorbate and aldarate metabolism**	**7 of 26**	**0.68**	**0.0424**
**hsa00830**<br>**Retinol metabolism**	**16 of 64**	**0.65**	**0.00028**
**hsa04742**<br>**Taste transduction**	**19 of 81**	**0.62**	**0.00020**
**hsa00980**<br>**Metabolism of xenobiotics by cytochrome P450**	**16 of 69**	**0.61**	**0.00045**
**hsa05204**<br>**Chemical carcinogenesis**	**17 of 75**	**0.6**	**0.00036**

Pathway	Description	count in network	strength	false discovery rate
hsa00590	Arachidonic acid metabolism	13 of 61	0.58	0.0055
hsa00982	Drug metabolism - cytochrome P450	13 of 64	0.56	0.0061
hsa04976	Bile secretion	16 of 89	0.5	0.0055
hsa04623	Cytosolic DNA-sensing pathway	11 of 62	0.5	0.0424
hsa04060	Cytokine-cytokine receptor interaction	38 of 282	0.38	0.00028
hsa04740	Olfactory transduction	51 of 420	0.33	0.00020
hsa04080	Neuroactive ligand-receptor interaction	37 of 330	0.3	0.0055

Reactome Pathways

Pathway	*description*	*count in network*	*strength*	*false discovery rate*
HSA-211935	Fatty acids	6 of 15	0.85	0.0495
HSA-156588	Glucuronidation	8 of 23	0.79	0.0182
HSA-445095	Interaction between L1 and Ankyrins	10 of 31	0.76	0.0064
HSA-912694	Regulation of IFNA signaling	8 of 25	0.75	0.0242
HSA-5576892	Phase 0 - rapid depolarisation	10 of 32	0.74	0.0075
HSA-1461957	Beta defensins			

HSA-933541<br>TRAF6 mediated IRF7 activation	10 of 34	0.72	0.0101
HSA-6805567<br>Keratinization	8 of 28	0.71	0.0440
HSA-1461973<br>Defensins	54 of 210	0.66	1.71e-14
HSA-420499<br>Class C/3 (Metabotropic glutamate/pheromone receptors)	11 of 43	0.66	0.0124
HSA-6809371<br>Formation of the cornified envelope	10 of 39	0.66	0.0216
HSA-6803157<br>Antimicrobial peptides	30 of 127	0.62	5.60e-07
HSA-211897<br>Cytochrome P450 - arranged by substrate type	20 of 87	0.61	0.00020
HSA-211945<br>Phase I - Functionalization of compounds	13 of 65	0.55	0.0216
HSA-1296071<br>Potassium Channels	19 of 105	0.51	0.0048
HSA-211859<br>Biological oxidations	18 of 103	0.49	0.0094
HSA-375276<br>Peptide ligand-binding receptors	32 of 214	0.42	0.00063
HSA-381753<br>Olfactory Signaling Pathway	28 of 193	0.41	0.0038
HSA-500792<br>GPCR ligand binding	50 of 391	0.36	0.00011

	56 of 455	0.34	8.31e-05
HSA-418555<br>G alpha (s) signalling events			
	64 of 532	0.33	3.36e-05
HSA-373076<br>Class A/1 (Rhodopsin-like receptors)			
	39 of 323	0.33	0.0041
HSA-112316<br>Neuronal System			
	46 of 406	0.3	0.0038
HSA-372790<br>Signaling by GPCR			
	125 of 1166	0.28	4.45e-08
HSA-388396<br>GPCR downstream signalling			
	115 of 1094	0.27	5.60e-07
HSA-418594<br>G alpha (i) signalling events			
	42 of 396	0.27	0.0184
HSA-1266738<br>Developmental Biology			
	105 of 1087	0.23	8.31e-05
HSA-162582<br>Signal Transduction			
	195 of 2741	0.1	0.0440
