# Supplementary material for: Vitamin D inhibits osteosarcoma by reprogramming nonsense-mediated RNA decay and SNAI2-mediated epithelial-to-mesenchymal transition
Source: Front Oncol. 2023 May 9;13:1188641. doi: 10.3389/fonc.2023.1188641 (PMC10203545; doi:10.3389/fonc.2023.1188641)
Supplement: Supplementary file 17 [file Table_1.docx]

**Table S1. qPCR primers (human)**

**Target gene BP Forward (5’-3’) Reveres (5’-3’) Reference**

CYP24A1 188 TGGCTTCAGGAGAAGGA AAA ACCAGGGTGCCTGAGTGTA G NM_000782. 5

VDR 192 GACCTCACCTCTGAGGA CCA GAACTTGATGAGGGGCTCAA CCDS55820

SOD2 213 CTGGACAAACCTCAGCC CTA CCTTGCAGTGGATCCTGATT CCDS83141

ACTB 198 GCAAAGACCTGTACGCC AAC ACATCTGCTGGAAGGTGGAC NM_001101. 5

SMG5 205 TGGCATCTTCGTCAGCATTG GACGAGGTAGGGAGACATGG ENST00000361813.5

SMG6 186 TGCCCCTCATCGTGATCAAT ATTCGAGTTCATTGCCACGG ENST00000263073.11

UPF2 186 GCTGCAAATCACTGGAACCA CTTCGGATGTTGGTAGCGAG ENST00000357604.10

STC2 184 GCTCCATCTTGAGCTTCTGC CTCTTGCTACCTCGCTCACC NST00000265087.9

FKBP5 195 AGCCAAGGGTGACTTTGAGA GAAGTCTTCTTGCCCATTGC ENST00000357266.9

SNAI2 204 TCGGACCCACACATTACCTT TTGGAGCAGTTTTTGCACTG ENST00000020945.4

CD44 215 CAGGGGTGTACATCCTCACA CAGGGGTGTACATCCTCACA ENST00000263398.11

MMP3 192 CCTCAGGAAGCTTGAACCTG GGGAAACCTAGGGTGTGGAT ENST00000299855.10

GILZ (TSC22D3) 183 ACCCCTGCTACCTGATCAAC TCTCCACCTCCTCTCTCACA ENST00000372383.9

DDIT4 200 CCTGGACAGCAGCAACAGT TACCAACTGGCTAGGCATCA ENST00000307365.4

HPRT 193 TGATGAAGGAGATGGGAGGC CCACCAATTACTTTTATGTCCCC ENST00000298556.8
